# Simulation-Based Education in the Training of Newborn Care Providers—A Malaysian Perspective

**DOI:** 10.3389/fped.2021.619035

**Published:** 2021-02-11

**Authors:** Kwai-Meng Pong, Jerrold Tze-Ren Teo, Fook-Choe Cheah

**Affiliations:** ^1^Pediatrics Department, Penang Adventist Hospital, Penang, Malaysia; ^2^Department of Pediatrics, Faculty of Medicine, Universiti Kebangsaan Malaysia, Kuala Lumpur, Malaysia

**Keywords:** simulation-based training, low-fidelity simulation, procedural skills, lay caregivers, debriefing, neonatal resuscitation, *in situ* simulation, interprofessional team

## Abstract

Simulation-based education (SBE) is increasingly used as an education tool to improve learning for healthcare providers. In newborn care practice, SBE is used in the Neonatal Resuscitation Program (NRP) and training in procedural skills. The NRP is a mandatory course in Malaysia for all house officers (interns) and medical officers (residents) during their pediatric rotation. Almost 30,000 of NRP providers have been trained over the last 5 years. The recent establishment of the Allied Healthcare Center of Excellence (AHCoE), an organization dedicated to promoting SBE, and Malaysian Society for Simulation in Healthcare (MaSSH) aims to enhance the integration of SBE into the healthcare training curriculum and set up a local healthcare simulation educator training program. Our experience in implementing SBE necessitated that we made several important choices. As there was no strong evidence to favor high-fidelity over low-fidelity simulation, and because simulation centers can be very costly to set up with limited resources, we chose SBE mainly in the form of low-fidelity and *in situ* simulation. We also identified an important developmental goal to train Malaysian instructors on structured debriefing, a critical activity for learning in SBE. Currently, debriefing is often carried out in our centers at an *ad hoc* basis because of time limitation and the lack of personnel trained. Finally, we aim to implement SBE further in Malaysia, with two axes: (1) the credentialing and recertification of physicians and nurses, and (2) the education of lay caregivers of high-risk infants before discharge from the neonatal intensive care unit.

## Introduction

In the past 10 years, simulation-based education (SBE) has been increasingly used as an educational tool to improve learning for healthcare providers. SBE is defined as an array of structured activities that represent actual or potential situations in education and practice. These activities allow participants to develop or enhance their knowledge, skills and attitudes, or to analyze and respond to realistic situations in a simulated environment (1, 2). Realistic scenarios in SBE aim to achieve “suspended disbelief” among trainees by replicating real-life situations with high fidelity (3). The anesthesia community first adopted SBE for medical training by using manikins in basic life support training (4, 5). Successful use of SBE has been reported subsequently in other specialties (e.g., emergency medicine, pediatrics, obstetrics and gynecology) (6–10). When SBE is implemented by experienced healthcare educators, it allows learning from mistakes, safe experimentation, deliberate practice, and standardized assessment of competency (11). With the increasingly higher number of medical graduates produced in countries such as Malaysia, there is as such, a declining opportunity for real-life neonatal resuscitation experiences for trainees^1^. Consequently, a new paradigm shift from the traditional “see one, do one, teach one” situations to using medical simulation in learning new skills and achieving competency has emerged.

This review discusses the application of simulation in teaching neonatal resuscitation and procedural skills as the core perspective in the development and promotion of SBE in neonatal practice in Malaysia.

## The Introduction and Development of a National NRP

In September 1996, the Department of Pediatrics of the Universiti Kebangsaan Malaysia (UKM), the Ministry of Health (MOH) of Malaysia and the Perinatal Society of Malaysia (PSM), officially introduced the Neonatal Resuscitation Program (NRP) in this country (12). To ensure a successful dissemination of the program nationwide, the NRP textbook, instructors' manual and test questions were translated into the local Malay language before the Malaysian NRP was launched. Professor Ronald Bloom from the USA was invited to Kuala Lumpur in 1996 to train the first batch of six core NRP instructors. These six instructors trained another 31 doctors and 6 nurses from all of the 13 states in Malaysia. Textbooks, instructor manuals, test questions, and manikins were then distributed to these core instructors for them to initiate the NRP training in their respective home states.

Dissemination of the NRP in Malaysia during the first 2 years was very encouraging. Of the first batch of 37 core instructors, 35 (94.6%) carried out training courses in their respective home states. This resulted in 513 new instructors and 2,256 providers who were trained and certified among health personnel from all over the country (13). Currently, there are 576 active instructors in Malaysia and a total of 29,152 NRP providers trained over the last 5 years (Table 1). The PSM is a professional non-governmental organization (NGO) that has taken the initiative to monitor the teaching activities of the NRP instructors as well as being engaged in the re-certification of providers (personal communication with Dr. See KC, director of NRP and affiliated to PSM).

**Table 1:** NRP-trained providers in relation to Malaysian annual live birth, perinatal mortality, and neonatal mortality rates during two different decades.

**Year**	**Number of trained providers^**^β^**^**	**Annual live birth^**^∧^**^**	**Perinatal mortality rate^**^+^**^ (per 1,000 live birth)**	**Neonatal mortality rate^**^+^**^ (per 1,000 live birth)**	**Asphyxia mortality rate^**^+^**^ (per 1,000,000 live birth)**	**Preterm delivery^**^#^**^ (Percentage of total deliveries)**
1996^*^	198	544,302	9.1	6.0	na	na
1997^*^	1,567	540,486	9.0	6.0	na	na
1998^*^	2,064	524,696	7.9	5.2	na	na
1999^*^	1,940	521,870	7.3	4.5	na	na
2010	na	491,239	7.7	4.3	4.4	8.1
2011	na	511,594	7.6	4.2	4.0	10.4
2012	na	508,774	7.3	4.0	1.9	11.3
2013	na	503,914	7.3	4.0	2.3	22.1
2014	na	511,865	7.2	3.9	2.0	21.9
2015	na	521,136	7.7	4.3	1.5	12.4
2016	4,362	508,203	8.3	4.2	1.1	11.7
2017	5,906	508,685	8.7	4.4	0.8	8.9

*NRP, Neonatal Resuscitation Program; na, not available*.

* *Data from Boo et al. (14)*.

β From the database of the Perinatal Society of Malaysia.

∧ *Data from the Department of Statistics, Malaysia^2^*.

+ *Data from the Ministry of Health, Malaysia^3^*.

# *Data from the National Obstetrics Registry, Malaysia^4^*.

This being a joint-venture effort by an NGO and the university, backed by a government agency (the MOH of Malaysia), showed that it is possible to successfully develop a locally customized NRP based on the original educational materials from an external source, in this case, the American Academy of Pediatrics. With the Malaysian NRP, it is encouraging to note that this SBE program goes in tandem with the improvement of health indicators of this country. The birth asphyxia-related mortality rate has declined to less than one in a million; perinatal mortality rate (PMR) and early neonatal mortality rate (NMR), have declined and stabilized to about eight and four in 1,000 live births, respectively, despite a rising preterm birth rate which doubled in 2013 and 2014. The Malaysian NRP is adjudged to have a positive impact on perinatal and neonatal care of this country (14). For the continuity of this quality improvement intervention, the NRP is a mandatory course funded by the government for all the pediatric house officers and medical officers in Malaysian public and teaching hospitals (15).

## Integrating SBE in the NRP Training

The earlier versions of NRP training consisted of didactic lectures, videos and skill stations, at which trainees practiced procedural skills on manikins. With this approach, the retention of knowledge and skills among the trainees reportedly lasted for only 6 months (16). The latest version of NRP integrated SBE with emphasis on team performance and behaviors during neonatal resuscitation. It utilizes a multiple learning approach, i.e., online testing, online case-based simulations, practical case-based simulation and debriefing which focus on key behaviors such as communication, critical leadership, and team-work skills (12). Some groups further showed significant improvement in confidence and performance levels among the learners who had a combined traditional and simulation based NRP course compared with the traditional alone. There was apparent improved teamwork and technical skills among the team members in high-fidelity simulation neonatal resuscitation in the delivery suites (17–20). Of note, a study reported reduction in the incidence of hypoxic-ischemic encephalopathy from 37.3 to 13.6 per 10,000 births after a simulation-based training for the perinatal team (21). This highlights the potential benefit of simulation-based training on patient outcomes. Similarly, the Malaysian NRP is deemed to positively impact perinatal-neonatal health outcomes of this country. The Malaysian NRP follows closely the updated versions of its original predecessor.

## High-Fidelity Simulation vs. Low-Fidelity Simulation

Fidelity is the principle of simulating a situation to realistically imitate true physiological realism. High-fidelity simulation (HFS), defined as simulation experiences that are extremely realistic and provide high level of interactivity and realism for the learner using technically sophisticated and computerized simulators manikins, has been used to enhance resuscitation education. On the other hand, low-fidelity simulation (LFS) is defined as simulation training that does not need to be controlled or programmed externally for the learner to participate; examples include case studies, role playing, or task trainers used to support students or professionals in learning a clinical situation or practice (2). The context of high- or low-fidelity may be viewed critically as simulation environments may also influence the learning and behavior change of the student.

Residents trained with high-fidelity simulators in neonatal resuscitation performed better in written test scores and a shorter duration taken to achieve successful intubation (22). However, there was no difference between the high- and low-fidelity training in terms of NRP performance score and the resuscitation duration. A randomized trial further demonstrated that there was no significant difference in the performance scores between low-fidelity and high-fidelity in NRP training (23).

Based on these, there is no strong evidence as yet to recommend the use of HFS over LFS. The cost of HFS may be overbearing to many centers in the developing world. There are only a few institutions in Malaysia that have the resources and are equipped with high-fidelity simulators. In a recent survey conducted informally through a neonatal simulation network in Malaysia, only five out of 32 (15.6%) hospitals run HFS. These centers are SBE-dedicated establishments such as the Allied Healthcare Center of Excellence (AHCoE), and several academic teaching hospitals. Those centers that do not run HFS quoted financial constraint as the main obstacle and more than three-quarter would like to have HFS made available with the opinion that this modality may enhance outcomes. Even so, it is important to emphasize that low-fidelity training can be as effective as HFS and the focus should be on the learning objectives and choosing a simulation modality that best meets those needs. The majority of Malaysian centers are still opting for low-fidelity manikins in their resuscitation training programs.

## *In Situ* Simulation in the Training of Providers

Recently, *in situ* simulation has become a popular form of SBE. In contrast to a dedicated simulation center, *in situ* simulation is held in the actual patient care setting in an effort to achieve a high level of fidelity and realism; for example, in the NICU, ambulance, small aircraft, or catheterization lab. This training is valuable to assess, troubleshoot, or develop new system processes (2).

It allows team members to train in handling rare complex events, to evaluate their team dynamics, and to assess the hospital and departmental policies/procedures in real locations and in real time. It may help to reveal latent safety issues in their actual work environment for potential quality improvement (24–26).

A study has shown significant improvements in teamwork and technical skills of staff members in initial neonatal resuscitation in the labor room after an *in situ* session. The median technical score and the median team score were significantly higher for the scenarios run, with a significant reduction in the number of hazardous events and an improvement in achieving the targeted heart rate (20). Furthermore, an unannounced mock drill in an *in situ* simulation was reported to improve the observance and performance of adopted best practices and self-confidence among residents during neonatal resuscitation (27).

As the cost of setting up is relatively cheaper compared to that of a simulation center, *in situ* simulation is an attractive SBE model for Malaysia and other developing countries. In a recent poll of 32 Malaysian hospitals, almost 80% stated that SBE for newborn care providers was delivered *in situ* at the neonatal ward or NICU. Only six centers run their simulation in a simulation center (unpublished data).

## Procedural Skills Training

In neonatal practice, an important component of training is in procedural skills. This is essential in achieving competency prior to performing on the real patient especially when dealing with risky procedures (28–30). In recent years, unlike in resuscitation training programs, HFS has proved to be an effective tool in teaching the skills of airway management, and served as an assessment tool for competency in airway management among pediatric residents (31). Some researchers have shown that using an infant chest tube insertion model that they created, there is a significant improvement in skill scores, knowledge and confidence after SBE together with retention of skills even after 1 month (32). Moreover, the Accreditation Council for Graduate Medical Education (ACGME) has accepted competence level of trainees on certain procedural skills such as intraosseous and umbilical catheter placement which are done in a simulated setting (33).

Currently, the procedural training for medical and nursing students, pediatric residency trainees in Malaysia infrequently utilizes manikins. A few simulation centers in the country are in the process of organizing bootcamps/workshops for procedure skill training, especially for those in pediatric residency programs. The COVID-19 pandemic is also making many teaching institutions to re-strategize in investing more heavily in SBE, obtaining high-fidelity manikins and task trainers even for assessments.

## Inter-Professional Team Training

Neonatology is a very good example of crisis resource management (CRM) where obstetricians, neonatologists, midwives, neonatal nurses work together to provide the best possible care for the baby, usually in stressful situations. Moreover, with the exposure to CRM, there was an overall improved trainees' self-perception, which directly led to an improvement in the time taken to initiate critical steps in pediatric resuscitation (pulse check, calling for help, setting intravenous access and placement of chest leads) (34, 35). The above studies suggest that CRM training in SBE may be effective in promoting the behaviors that affect the outcomes in resuscitation.

The latest version of NRP has included emphasis on CRM by incorporating the 10 key behavioral skills and team performance into the revised syllabus (36). Local unpublished data indicated that almost half of the 32 Malaysian NICUs surveyed, run interprofessional team (IPT) training. Even so, up to three quarter of these centers still carried out SBE separately for a particular profession only, for example a NRP provider training course may be entirely subscribed by nurses. Although we are moving toward promoting more IPT in SBE, in many situations there may be a need to cater to a particular category of healthcare professional because of an overwhelming response. In some cultures, the lack of assertiveness in communicating may be an impediment to IPT training. Hence, there may be some necessary modifications to cater to the local context to enhance team involvement in the training for CRM.

## Debriefing

Debriefing is considered to be a crucial component of SBE. The learners will have the opportunity to recover from the stressful scenarios, make sense of the simulation activities, reflect and evaluate their performance to change the way they think and practice to consolidate their learning. A systematic review revealed that simulation with debriefing has a favorable effect on learning outcomes when compared with no intervention (37).

Neither video-assisted nor oral debriefing solely conferred any extra benefit. There was, however, significant improvement in the NRP performance scores in both groups after debriefing (38). A personal observation by the first author of this article, was that nurses in Malaysia were generally less communicative during the SBE sessions he conducted. Video-assisted debriefing may be useful in such situations, with trained simulation educators playing back the video to highlight some essential learning points.

In a recent local survey, only slightly more than half of the 32 centers hold a debriefing session after each SBE. Time limitation is also a factor in carrying out debriefing especially in centers that are busy with a heavy workload and lacking in staff. Of the 13 NICUs that organized “train-the-trainer” simulation educator course, only six hold a debriefer course. Effective debriefing needs simulation educators who have the skills and knowledge in conducting debriefing, such as those who are Certified Healthcare Simulation Educator^Ⓡ^ (CHSE^Ⓡ^). Some reports indicated that structured debriefing after handling the management of cardiac arrest, accelerated the return of spontaneous circulation and improved neurologic outcome of real patients (39, 40). As such, there is immense potential of debriefing as a useful educational and quality improvement tool. There are currently three CHSE^Ⓡ^ trainers in Malaysia who plan to hold more local courses that focus on the training to perform structured debriefing.

## SBE in the Training of Professional and Lay Providers

The use of simulation-based training has been widely adopted in the USA, with 81% among the respondents of the Neonatal-Perinatal Medicine fellowship programs reportedly used simulation (41).

Simulation-based training/assessment is gradually incorporated into the pediatric residency training programs in Malaysia, as trainees are required to undergo successfully and be certified as providers in NRP and Pediatric Advanced Life Support (PALS), respectively.

**Figure 1:**
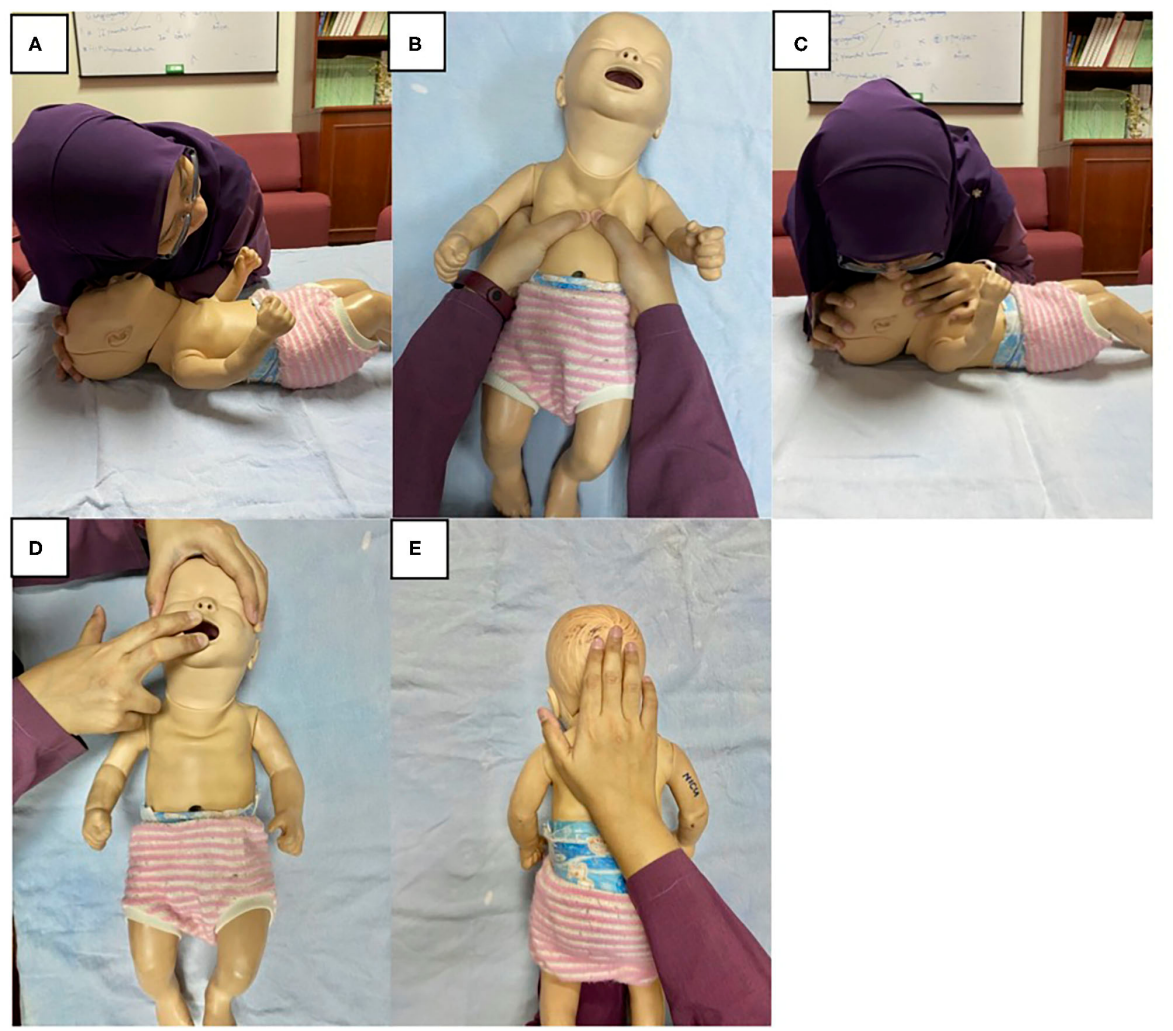
Basic life support simulation training for lay caregivers of infants before discharge from the NICU. The step-by-step instruction when encountering an unresponsive infant includes, **(A)** listen for breathing, **(B)** perform chest compression, **(C)** give rescue breaths. In an infant suspected of choking, **(D)** opening mouth to look for and clear any foreign objects, **(E)** perform back blows. Written informed consent was obtained from the individual for the publication of any potentially identifiable images included in this article.

In family-centered care, the involvement of parents or the caregiver is crucial before a high-risk neonate is discharged from the NICU^5^. The transition from NICU to home often results in much anxiety to the family. SBE may mitigate such a stressful period. Scenarios mimicking clinical conditions involving the infant can be simulated using various models and manikins. Family members are then encouraged to participate in multiple simulation sessions paced accordingly over a period of time, with them attending to various scenarios which include life-threatening conditions (42). With SBE, caregivers are more confident and prepared to handle emergency situations and resuscitation. Consequently, they feel more ready and confident in creating a safer environment for their infant (43). In Malaysia, more than half of the surveyed NICUs have healthcare providers entrusted to routinely educate caregivers regarding infant care and safety when at home (local unpublished data). Before discharge, important topics relating to responses to common emergencies and basic life support are relayed utilizing simulation with the appropriate models and manikins (Figure 1).

## Recent Developments and Addressing Challenges in SBE

The SBE is faced with challenges that include limited resources and the lack of qualified or trained instructors. Trained healthcare simulation educators are mostly based in the university and tertiary referral hospitals. Recently, the UKM Medical Center in Kuala Lumpur has become a pacesetter when it became an authorized international training center for the AHA-sanctioned resuscitation programs. There is an increasing need for universities to lead in setting up simulation centers for training and assessment of competencies in resuscitation and procedural skills. A survey on the academic healthcare institutions in Malaysia identified the common challenges as financial support, insufficient of trained faculty and lack of available facilities. Most faculty staff had attended technical training, but there was still little training and development courses for educators opting for this career advancement pathway. Utilization of resources was also limited for research (44).

The AHCoE is an institution that originated as a not-for-profit organization. It was set up with a grant from the Malaysian Federal Government to create a regional shared network of educators dedicated to improve patient care^6^. Established in March 2010, the mission of the center includes effective integration of SBE. The establishment of the Malaysian Society for Simulation in Healthcare (MaSSH) signifies the country's further commitment to advance healthcare simulation training. The MaSSH was founded in 2016 and recently the society has collaborated with other healthcare simulation societies across the region, including the Society for Simulation in Healthcare (SSH) and Pan Asia Simulation Society in Healthcare (PASSH)^7^. In the pipeline is initiating the certification of healthcare simulation educators in Malaysia to ensure the provision of high-quality SBE. For a start, the SSH has just established a center for CHSE exam in Kuala Lumpur in 2020.

Collaborative efforts with NGOs also saw three neonatal emergency simulation (NESim), “train-the-trainers” workshops being organized by the MOH and PSM. NESim is a one- day simulation workshop with the participants handling real life neonatal emergency conditions and learning to perform optimally in stressful circumstances using manikins. The workshop comprised of interactive lectures and group learning activities, which include simulation scenarios and debriefing. A total of 54 participants were trained so far and these trainers were then to carry out training of their own staff in their respective hospitals. The AHCoE is also in the process of developing a Neonatal Simulation Educator (Train-the-Trainer) course in collaboration with the MaSSH. A mentorship program is also planned to guide the novice simulation educator who just qualified.

## Conclusion

Collaboration between the university and NGO created the impetus to initiate the NRP in Malaysia. Positive healthcare outcomes were seen consequently with major stakeholders commenced funding of simulation-based training in neonatal resuscitation, utilizing low-fidelity and *in situ* simulation for newborn care providers. SBE is increasingly becoming a tool for assessment of competency and a requirement for credentialing in specialist board registration and practice recertification.

## Data Availability Statement

The original contributions presented in the study are included in the article/supplementary material, further inquiries can be directed to the corresponding author/s.

## Ethics Statement

Written informed consent was obtained from the individual for the publication of any potentially identifiable images or data included in this article.

## Author Contributions

K-MP prepared the first draft of this manuscript, created the survey, analyzed and collated the data, and statistics from the Perinatal Society of Malaysia. JT-R contributed in the writing, created the table and figure, accessed the statistics from sources, ensured editorial accuracy of the manuscript, and reference compilation. F-CC steered the project, critically edited, primarily involved in re-structuring, and re-writing and re-formatting the manuscript into the final form. All authors listed have made a substantial, direct and intellectual contribution to the work, and approved it for publication.

## Conflict of Interest

K-MP received honorarium for teaching activities in workshops conducted by the Allied Healthcare Centre of Excellence, (AHCoE), Penang, Malaysia. The remaining authors declare that the research was conducted in the absence of any commercial or financial relationships that could be construed as a potential conflict of interest.

## Footnotes

^1^Government to resolve issue of housemen placement. Available online at: https://www.nst.com.my/news/nation/2018/06/378286/government-resolve-issue-housemen-placement (accessed June 19, 2020).

^2^Department of Statistics Malaysia Official Portal. Available online at: https://www.dosm.gov.my (accessed December 30, 2020).

^3^Petunjuk Kesihatan. Malaysia Ministry of Health. Available online at: https://www.moh.gov.my/index.php/pages/view/58 (accessed December 30, 2020).

^4^National Obstetrics Registry Annual Report. National Obstetrics Registry Malaysia. Available online at: http://www.acrm.org.my/nor/ (accessed December 30, 2020).

^5^The Eight Principles of Patient-Centered Care Oneview. Available online at: https://www.oneviewhealthcare.com/the-eight-principles-of-patient-centered-care/ (accessed September 16, 2020).

^6^Allied Healthcare Center of Excellence. Available online at: https://ahcoe.my/ (accessed June 22, 2020).

^7^About Us - MaSSH. Available online at: https://massh.org.my/about-us/ (accessed June 22, 2020).
